# Plant-Derived Bioactive Compounds for Pharmacological Applications

**DOI:** 10.3390/ijms27156867

**Published:** 2026-07-31

**Authors:** Ilian Badjakov, Ivayla Dincheva

**Affiliations:** Department of Agrobiotechnologies, Agrobioinstitute, Agricultural Academy, 8 Dragan Tsankov Blvd., 1164 Sofia, Bulgaria

Plant-derived bioactive compounds continue to attract considerable scientific interest because of their structural diversity and capacity to influence multiple molecular pathways. Their pharmacological relevance extends to inflammation, oxidative stress, cardiovascular disease, neurodegeneration, cancer, antimicrobial discovery, pharmaceutical delivery and nutrition. Research in this field is moving beyond descriptive phytochemistry towards a more integrated approach. Chemical characterisation, biological validation, mechanistic interpretation, formulation and sustainable production are increasingly considered together. The Special Issue, entitled “Plant-Derived Bioactive Compounds for Pharmacological Applications”, reflects this transition through investigations of macrophage lipid deposition [1], polyphenols in the central nervous system and their liposomal delivery [2], anticancer phenanthrenes from Juncus tenuis [3], the phytochemical profile of *Porophyllum gracile* [4], a curcumin nanocarrier [5], antimicrobial metabolites from basidiomycetes [6], the pharmacology of mollugin [7] and polyunsaturated fatty acid biosynthesis across aquatic trophic levels [8].

The investigations presented in the Special Issue represent complementary stages of contemporary research on natural products. They range from metabolite profiling and structural elucidation to cellular assays, pathway-oriented interpretation, drug-combination analysis, delivery-system development and biological production [1,2,3,4,5,6,7,8]. Although the principal focus is on plant-derived compounds, the inclusion of fungal metabolites and aquaculture-related bioactive lipids broadens the discussion to a wider natural-products framework [6,8]. Collectively, the eight contributions show that the scientific value of natural compounds depends not only on their chemical diversity. It also depends on linking that diversity to defined biological activities, technological solutions and translational opportunities.

A major message emerging from the Special Issue is the need to connect bioactivity with molecular mechanism. The study of syringin and phillygenin provides a clear example in the context of atherosclerosis-related macrophage biology [1]. Syringin, a phenylpropanoid glycoside isolated from *Syringa vulgaris*, and phillygenin, a lignan obtained mainly from Forsythia intermedia, were found to reduce cholesterol-induced lipid deposition in macrophages through distinct but complementary mechanisms. Syringin markedly decreased CD36 receptor expression through a PPAR-γ-dependent pathway and limited foam-cell formation. Phillygenin increased ABCA1 transporter expression through a HO-1-dependent pathway and promoted cholesterol efflux [1]. Such findings move phytochemical research beyond broad antioxidant or anti-inflammatory descriptions and place natural compounds within defined disease-related processes.

Mechanistic interpretation is further developed in the reviews on polyphenols and mollugin. Polyphenols in the central nervous system are presented as modulators of oxidative stress, neuroinflammation, microglial activation, neurogenesis and signalling pathways such as PI3K/Akt, MAPK, Nrf2 and CREB [2]. Their relevance to Alzheimer’s disease, Parkinson’s disease and multiple sclerosis is considered alongside major barriers to therapeutic application. These include poor solubility, instability, low bioavailability and restricted penetration across the blood–brain barrier [2]. The review on mollugin extends the mechanistic scope by describing this compound from *Rubia cordifolia* as a multifunctional agent involved in inflammatory, antioxidant, anticancer, neuroprotective, osteogenic and multidrug-resistance-related pathways [7]. Together, the two reviews underline a defining feature of modern natural-product pharmacology: bioactive metabolites are increasingly interpreted as regulators of complex biological networks rather than as agents producing isolated effects.

Discovery and structural characterisation remain essential to the field. This is demonstrated by the investigation of phenanthrenes from Juncus tenuis [3]. Five previously undescribed compounds, tenuins A–E, and fourteen known phenanthrenes were isolated and characterised using NMR spectroscopy and high-resolution mass spectrometry. Their antiproliferative activity was evaluated against doxorubicin-sensitive and doxorubicin-resistant human colorectal cancer cell lines, together with a non-tumourigenic fibroblast line [3]. Although the most active diphenanthrenes showed limited selectivity towards cancer cells, one compound displayed very strong synergism with doxorubicin in resistant COLO 320 cells [3]. The pharmacological significance therefore extends beyond cytotoxicity to the possibility of chemosensitisation and modulation of drug resistance.

The investigation of *Porophyllum gracile* provides a complementary example of how extensive chemical profiling can be connected with functional assays [4]. GC-MS, HPLC-DAD and GC-FID analyses identified more than 120 compounds in dried leaves and stems. These included fatty acids, chlorogenic acid derivatives, quercetin derivatives, terpenes, ketones, aldehydes and alcohols [4]. Extracts reduced intracellular reactive oxygen species in ARPE-19 cells exposed to doxorubicin-induced oxidative stress and, in some cases, showed greater activity than vitamin E. No antiproliferative activity was detected against the tested cancer cell lines, and no cytotoxicity was observed in non-cancerous cells [4]. The findings support the potential of P. gracile as a natural antioxidant source without overstating its anticancer relevance.

Pharmaceutical formulation represents another important direction within this Special Issue. Many promising phytochemicals are limited by low solubility, instability, rapid degradation or poor bioavailability. Two contributions address this challenge from complementary perspectives. The review on central nervous system polyphenols discusses liposomal and targeted nanocarriers as tools for improving delivery across biological barriers [2]. The Jamamina study provides an experimental example through a sustainable nanostructured lipid carrier containing curcumin and a natural deep eutectic solvent composed of malic acid and betaine [5]. The formulation, based on Amazonian tucumã butter and jambu oil, achieved high curcumin encapsulation efficiency and improved its solubility and colloidal stability. In L132 cells, Jamamina reduced TNF-α and IL-6, increased IL-10, suppressed MMP-2/9 activity and preserved cytoskeletal integrity under oxidative stress [5]. The translational value of natural compounds therefore depends not only on intrinsic bioactivity, but also on effective formulation, delivery and technological optimisation.

The antimicrobial and production-oriented dimensions are illustrated by the screening of basidiomycete strains [6]. Extracts from culture liquids and submerged mycelia of eighteen strains were evaluated using agar well diffusion assays, minimum inhibitory concentration determination and HPLC-MS profiling. Sixteen strains produced antimicrobial metabolites. Selected strains of *Hericium*, *Fomitopsis* and *Laetiporus* showed antibacterial or antifungal activity [6]. Preliminary HPLC-MS analysis identified hericerin in *Hericium erinaceus* and sulfureuine H in *Laetiporus sulphureus* [6]. Although fungal metabolites extend beyond the strict plant-derived category, their inclusion strengthens the broader natural-products perspective and highlights submerged cultivation as a practical route for antimicrobial metabolite production.

A wider nutritional and ecological perspective is provided by the review of polyunsaturated fatty acid biosynthesis across three trophic levels in freshwater aquaculture [8]. The article examines the production, regulation and transfer of EPA and DHA among microalgae, zooplankton and fish larvae. It links molecular biosynthesis with nutrition, trophic transfer, aquaculture sustainability and food-chain quality [8]. Bioactive molecules are therefore considered not only as isolated therapeutic candidates, but also as components of biological networks that influence health, resource management and sustainable production.

Taken together, the investigations presented in this Special Issue trace a shared research sequence. It begins with biodiversity-driven discovery and chemical characterisation. It progresses through biological validation and mechanistic interpretation. It then extends towards pharmaceutical optimisation, antimicrobial discovery and sustainable production. Each contribution represents a distinct stage within that sequence [1,2,3,4,5,6,7,8]. The principal message is clear: the future relevance of plant-derived and related natural bioactive compounds will depend on the integration of phytochemistry, molecular pharmacology, formulation science, biotechnology and sustainable production.
